# Challenges and best practices for digital unstructured data enrichment in health research: A systematic narrative review

**DOI:** 10.1371/journal.pdig.0000347

**Published:** 2023-10-11

**Authors:** Jana Sedlakova, Paola Daniore, Andrea Horn Wintsch, Markus Wolf, Mina Stanikic, Christina Haag, Chloé Sieber, Gerold Schneider, Kaspar Staub, Dominik Alois Ettlin, Oliver Grübner, Fabio Rinaldi, Viktor von Wyl, for the University of Zurich Digital Society Initiative (UZH-DSI) Health Community

**Affiliations:** 1 Digital Society Initiative, University of Zurich, Zurich, Switzerland; 2 Institute for Implementation Science in Health Care, University of Zurich, Zurich, Switzerland; 3 Institute of Biomedical Ethics and History of Medicine, University of Zurich, Zurich, Switzerland; 4 Center for Gerontology, University of Zurich, Zurich, Switzerland; 5 CoupleSense: Health and Interpersonal Emotion Regulation Group, University Research Priority Program (URPP) Dynamics of Healthy Aging, University of Zurich, Zurich, Switzerland; 6 Department of Psychology, University of Zurich, Zurich, Switzerland; 7 Epidemiology, Biostatistics and Prevention Institute, University of Zurich, Zurich, Switzerland; 8 Department of Computational Linguistics, University of Zurich, Zurich, Switzerland; 9 Institute of Evolutionary Medicine, University of Zurich, Zurich, Switzerland; 10 Center of Dental Medicine, University of Zurich, Zurich, Switzerland; 11 Department of Geography, University of Zurich, Zurich, Switzerland; 12 Dalle Molle Institute for Artificial Intelligence (IDSIA), Switzerland; 13 Department of Quantitative Biomedicine, University of Zurich, Zurich, Switzerland; 14 Fondazione Bruno Kessler, Trento, Italy; 15 Swiss Institute of Bioinformatics, Switzerland; University of the Philippines Manila, PHILIPPINES

## Abstract

Digital data play an increasingly important role in advancing health research and care. However, most digital data in healthcare are in an unstructured and often not readily accessible format for research. Unstructured data are often found in a format that lacks standardization and needs significant preprocessing and feature extraction efforts. This poses challenges when combining such data with other data sources to enhance the existing knowledge base, which we refer to as digital unstructured data enrichment. Overcoming these methodological challenges requires significant resources and may limit the ability to fully leverage their potential for advancing health research and, ultimately, prevention, and patient care delivery. While prevalent challenges associated with unstructured data use in health research are widely reported across literature, a comprehensive interdisciplinary summary of such challenges and possible solutions to facilitate their use in combination with structured data sources is missing. In this study, we report findings from a systematic narrative review on the seven most prevalent challenge areas connected with the digital unstructured data enrichment in the fields of cardiology, neurology and mental health, along with possible solutions to address these challenges. Based on these findings, we developed a checklist that follows the standard data flow in health research studies. This checklist aims to provide initial systematic guidance to inform early planning and feasibility assessments for health research studies aiming combining unstructured data with existing data sources. Overall, the generality of reported unstructured data enrichment methods in the studies included in this review call for more systematic reporting of such methods to achieve greater reproducibility in future studies.

## Author summary

The digital revolution has led to an exponential growth of novel sources of data, such as data from social media or wearables. These data are mainly unstructured, which means they are not available in a pre-defined format that is easy to analyze. Digital unstructured data present an unprecedented opportunity for health researchers to enrich the existing knowledge base for studies and contribute to personalized and evidence-based medicine. We reviewed literature to summarize challenges that researchers commonly encounter and their possible solutions for combining digital unstructured data with other data sources in health research. The novelty and large availability of digital unstructured data are connected with two overarching barriers and challenges. First, digital unstructured data require novel forms of processing and standardization. Second, there is a lack of standardized guidelines, tools or techniques analyzing and incorporating them in research. Our review provides guidance for initial research planning aimed at researchers who wish to apply digital unstructured data enrichment in their studies, and best practices to overcome such challenges through a feasibility assessment.

## Introduction

Digitalization has resulted in the generation of a broad variety of data with valuable health-related information that can contribute to health research. Digital data in healthcare originate from a wide range of sources, from structured clinical data, such as laboratory test results or patient-reported outcome measures, to unstructured data, such as free text data, collected within or outside of a clinical setting [1]. This wealth of data holds great potential to advance health research and, ultimately, prevention, and patient care delivery. However, over 80% of digital data in healthcare is available as unstructured data [1], requiring new forms of data processing and standardizing that prove challenging to health researchers. The challenging nature of such data is also reflected in the fact that these data are often not specifically collected for research purposes (e.g., data from social media).

Unstructured data are commonly defined as data that are not readily available in predefined structured formats, such as tabular formats [2–5]. However, there is no unified, standardized definition of digital unstructured data in health research. In the literature, digital unstructured data are often referred interchangeably as “big data”, “digital data”, “unstructured textual data” and described as “high-dimensional”, “large-scale”, “rich”, “multivariate” or “raw” [1,4,6–10]. Digital unstructured data are a valuable source of information that may not be captured in structured data and can complement the knowledge base to enable data enrichment to further inform health research. For example, open-ended patient self-reports or smartphone data can be used to complement longitudinal laboratory, clinical, and survey data [11–13]. We refer to this combination of digital unstructured data with other data sources as digital unstructured data enrichment.

Digital unstructured data enrichment leverages real-time measurements and monitoring in natural living environments to gain insights into individuals’ lifestyles and behaviors, contributing to digital phenotyping [14] and better understanding of health risks or diseases [12]. Furthermore, it can contribute to a higher representation of under-researched population groups (e.g., ethnic minorities) [15] and provide a deeper understanding of participants’ daily life contexts over extended periods, as well as outside of clinical settings [16]. This wealth of combined data can foster personalized and adaptive health assessments in real-time and promote inclusivity of under-researched population groups in health research.

Despite the opportunities presented by the abundance of digital unstructured data in advancing health research, methodological challenges remain due to the need for extensive preprocessing and meaningful combination with other data sources [3,4,12,17,18]. For example, researchers might struggle with identifying the most suitable methods to work with such data that are aligned with established best practices in research. This might lead to the generation of hypotheses based on available data rather than following the established scientific process of developing hypotheses and methods before the data is available [19].

These challenges with digital unstructured data can hinder their enrichment with other data sources that are often in a structured format. The challenges of combining structured data sources and associated approaches to overcome these challenges are well reported in the literature. For example, commonly reported challenges include biased analysis due to selective data availability or systematic errors in the linkage process. These can be overcome through appropriate study design, considering the quality of the data linkage and assessing systematic errors [20]. For unstructured data, however, the persisting challenges and methodological approaches to facilitate their combination with existing data sources are missing in the literature. Current studies place focus on the pre-processing or optimization of methods with digital unstructured data, rather than informing study planning for enabling digital unstructured data enrichment in health research. The additional complexity of digital unstructured data requires specific attention to understand the challenges that emerge through its combination with other data sources. As such, there is a need for interdisciplinary guidance based on standards and best practices to inform the planning and reporting of health research studies that incorporate digital unstructured data enrichment.

## Aims

This systematic narrative review aims to explore current research practice, standards and requirements to use digital unstructured data and their combination with existing data in the health areas of cardiology, neurology and mental health. Specifically, we aim to answer the following research question:

*How can health researchers enable the proper (systematic, reliable, valid, effective, and ethical) use of digital unstructured data to enrich the evidence base from available data sources*?

To answer this research question, this review 1) identifies and describes the main challenge areas associated with the use of unstructured data to enable digital unstructured data enrichment in the health areas of cardiology, neurology and mental health; 2) provides a summary of possible solutions for common challenges associated with digital unstructured data enrichment; 3) provides guidance for the initial assessment of whether the inclusion of unstructured data is a feasible and appropriate for the study intended research tasks.

We focused on the fields of neurology, cardiology and mental health. These were chosen due to the high data availability of unstructured data in these fields and their well-established use for research and healthcare [21,22]. Furthermore, these three fields reflect the expertise in the review team, and the findings are likely applicable to other disease areas. The goal of this review is to guide study planning and implementation of unstructured data use for data enrichment from a methodological perspective based on existing literature. As such, our approach aims to guide and enable applied researchers to practically apply unstructured data enrichment in health research.

## Methodology

### Definitions of unstructured data and digital unstructured data enrichment

We developed the following working definition for *digital unstructured data* in accordance with the literature: unstructured data are raw data that fulfill at least one of the following conditions: a) are not in a pre-defined structure (e.g., tables), b) and/or data that are not ready-to-use and require substantial pre-processing or feature extraction efforts to extract the desired information for analyses (e.g., free text data stored in table) [3,4,12,18]. Substantial pre-processing and feature extraction efforts refers to the process of transforming raw unstructured data into data that are ready-to-use, credible and meaningful for a research question and research project [23]. For example, unstructured sensor data might require signal processing techniques such as noise filtering, data interpolation or outlier removal for meaningful information extraction. We added the second condition to emphasize that digital unstructured data can exist either in its original unstructured format or within a structured format. For both cases, the data need significant pre-processing before they can be used, which adds complexity to the process of data enrichment. This is also reflected in the literature where sensor data or biosignal data, although available in a structured format, are considered to be unstructured data because of their associated high pre-processing efforts [1,24,25]. High pre-processing burden associated with our working definition of digital unstructured data has connections to the concept of big data. Big data is commonly defined by the 4V attributes (volume, velocity, variety, and veracity) [4,26,27], and applies to structured as well as unstructured big data. We consider unstructured big data to represent a subset of digital unstructured data.

We further define digital unstructured data enrichment as the use of digital unstructured data in combination with other data sources to augment the available evidence base and contribute to relevant domain knowledge in health research and clinical practice. We envision a situation where digital unstructured data are already available (e.g., clinician notes or collected wearable data), and researchers wish to utilize these digital unstructured data in combination with structured survey data from the same patients. Within this definition we also consider the complexity and challenges associated with digital unstructured data itself, which can create difficulties when linking it with other data sources.

### Included types of digital unstructured data

In this review, we consider text data, including unstructured data from electronic health records (EHR), unstructured big data, and sensor data from wearables and other devices, including electroencephalogram (EEG) as common sources of digital unstructured data. Despite their widespread use in health research, we did not consider imaging and video data in this review, as these data are often bound with additional technical challenges in the enrichment process that may not generalize to other unstructured data types [28,29].

### Search strategy

**Fig 1:**
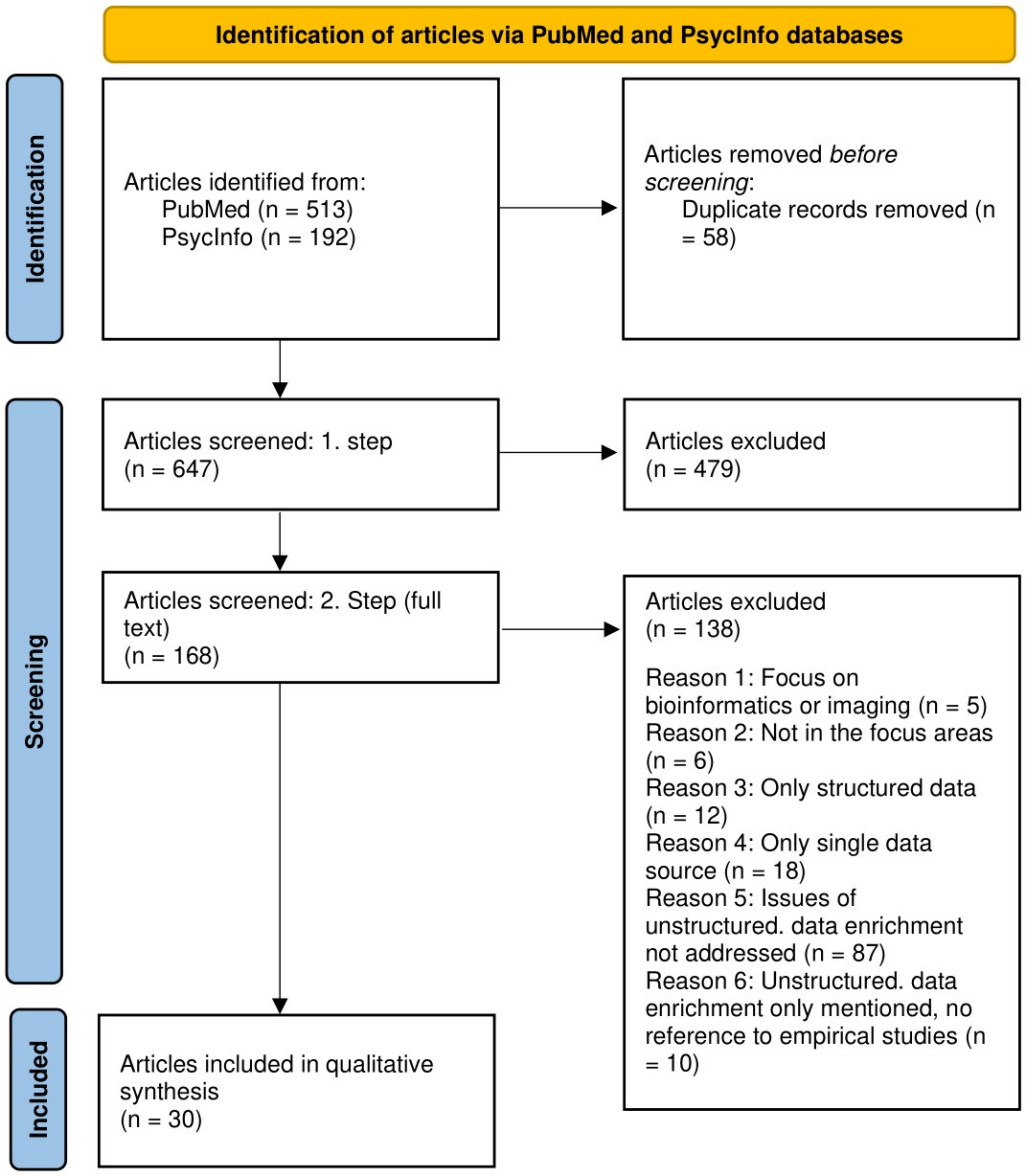
PRISMA flowchart.

We conducted a systematic narrative review guided by the Preferred Reporting Items for Systematic Reviews and Meta-Analyses (PRISMA) 2020 statement [30] (S1 PRISMA Checklist). Fig 1. PRISMA flowchart provides the flowchart for the screening and study selection process. Our study selection was guided by the inclusion and exclusion criteria displayed in Textbox 1 and Textbox 2, respectively. We performed our search on PubMed and PsycInfo for 1) general overview articles, 2) primary research articles, and 3) articles describing databases, all including relevant information on digital unstructured data enrichment. The complete search syntax including all keywords can be found in S1 Text.

**Textbox 1. Literature Review Inclusion Criteria**

1. Published, peer-reviewed articles from 2016–2021.
2. Articles written in English.
3. Articles from the field of neurology, cardiology, mental health, or focusing on one of the diseases listed in the keywords.
4. Articles mentioning various sources of unstructured data and structured data in one of the three defined health fields.
5. Articles discussing challenges, limitations, or gaps of the combination of unstructured data with other data sources in health research.

**Textbox 2. Literature Review Exclusion Criteria**

1. Articles focusing on imaging analysis or bioinformatics.
2. Articles outside of the three health-research areas: cardiology, neurology, mental health.
3. Articles including only structured data.
4. Articles leveraging a single data source (that is, no data enrichment).
5. Articles not addressing issues linked to the digital unstructured data enrichment.
6. Articles that are only broadly *mentioning* digital unstructured data enrichment in a theoretical way either without referring to specific examples from empirical studies or without providing practical approaches that can be applied further studies.
7. Systematic Reviews (narrative or literature reviews are included).
8. Protocols.

Screening was conducted in two phases. In the first step, we screened the titles and abstracts from the studies based on the inclusion criteria (Textbox 1). In the second step, we performed a full-text screening of the articles selected in the first step and excluded articles that matched the criteria outlined in Textbox 2. In both steps, one investigator (JS) assessed all articles and a second investigator (PD) performed checks on a randomly selected sample of articles for each screening phase. Any disagreements were discussed and, if required, a decision was achieved through the principal investigator (VvW).

### Data extraction and synthesis

Data extraction was systematic yet developed iteratively. The initial data extraction included study characteristics and attributes relevant to our research question. During the full-text screening, seven overarching topics related to digital unstructured data enrichment were identified and used for data extraction. The topics were the following: 1) medical field and subfield of the study, 2) main motivation for digital unstructured data enrichment, 3) data enrichment scope (e.g., gathering accurate information about disease severity), 4) type(s) of unstructured data, 5) limitations of unstructured data (e.g., quality/completeness), 6) challenges of digital unstructured data enrichment, and 7) proposed or discussed approaches for overcoming the mentioned challenges. The extracted data are to be found in S1 Data.

A narrative synthesis of the results was conducted to provide an overview of the challenges and proposed solutions related to digital unstructured data enrichment. This choice was also motivated by the heterogeneity of included studies that ranged from overview papers to original research studies. To address study aims 1 and 2 (i.e., description of common challenges and their possible solutions), the extracted study data on the topics 5 and 6 (i.e., limitations and challenges associated with enabling digital unstructured data enrichment) were grouped into challenge areas. The challenge areas include not only topics directly connected with data enrichment, but also related to the unstructured data use itself, as this is an essential requirement to enable digital unstructured data enrichment. For each challenge area, relevant possible solutions to tackle the challenges were summarized. For study aim 3 (i.e., providing guidance), we developed a preliminary checklist based on findings from our literature review to guide early study planning and feasibility assessment steps for studies that aim to include unstructured data in their methodology. To this end, the identified challenge areas from study aim 1 were reformulated into checklist questions and ordered according to the common study planning stages in health research [31]. Finally, the checklist was complemented and refined based on domain-specific expertise represented by the interdisciplinary team.

## Results

Our database search yielded 705 articles (Fig 1). Overall, 30 articles were included for assessment in this review.

### General description of included studies

A description of the 30 included articles [3–5,8–13,16,18,32–50] is presented in S1 Table. The most frequently discussed types of unstructured data sources in the selected articles were electronic health records (n = 13) and sensor data (n = 7). The most commonly cited motivations for digital unstructured data enrichment were to include more objective measures in their research, for example, to improve understanding of disease mechanisms and disease prediction, and to strengthen the existing evidence base in precision medicine, real-time monitoring, and real-world data collection.

The most prevalent challenge areas in enabling digital unstructured data enrichment were: 1) the lack of meta-information for unstructured data (n = 6), 2) standardization issues (n = 21) 3) data quality and bias in data (n = 13), 4) infrastructure and human resources (n = 12), 5) finding suitable analysis tools, methods and techniques (n = 15), 6) alignment of unstructured data with a research question and design (n = 11), as well as 7) legal and ethical issues (n = 11). These challenges span across all study stages involving data in a health research study: from data collection to data interpretation. Definitions of the main challenge areas and a brief explanation of their relevance for health research are given in S2 Table.

### Challenge areas

In the next sections, we summarize the seven identified challenge areas associated with enabling digital unstructured data enrichment in the field of cardiology, neurology and mental health and the proposed possible solutions to address them.

*1*. *Lack of meta-information for unstructured data*

CHALLENGES: Lack of meta-information (e.g., describing data structure and properties or sample population) has been acknowledged as an obstacle for unstructured data findability, integration, interchangeability, and interpretation [3,5,8,18]. Insufficient meta-information might limit the translation of a study’s findings into clinical practice [3,8] as important contextual information, such as information on the time in which the data were collected might be missing to assess the usability and correct interpretation of data and, consequently, their combination with other data sources [3].

POSSIBLE SOLUTIONS: Proposed possible solutions included the standardization of meta-information (e.g., through a standardized format for meta-information through open science standards), which may also resolve issues of data interpretation and their alignment with research questions and designs [3–5,18,43]. Specifically, one suggestion was to provide information for four important aspects in each study: subjects included in the data, context of collection, observations, and time of data collection [3]. Moreover, a greater availability of standardized meta-information was suggested, as this would facilitate the determination of suitable specific unstructured data for a given research question and their linkage with other data sources [3,4].

*2*. *Standardization issues*

CHALLENGES: The most frequently discussed challenge (n = 21) was the lack of a standardized framework for the description of disease phenotypes (e.g., symptoms, clinical presentation), as well as a lack of commonly defined terminologies, ontologies, and data labels [3–5,8–10,13,16,18,32–34,36,37,43,45,48,50]. For example, different terms may be used for a seizure with alteration of consciousness by different physicians [32] or for the administration of a specific dose of a given drug [33]. These issues are particularly prevalent in EHRs or clinical annotations [18] where, for example, terminologies and phenotyping may differ across healthcare settings or change over time [4,32,38]. This poses a notable challenge for data linkage because relevant features might vary in the different data sets and prevent their proper linkage. There is also an observed lack of standardized data management methods [8,45] and regulatory standards to guide and assess the use of novel technology and their associated unstructured data in clinical applications such as clinical trials [3,12].

POSSIBLE SOLUTIONS: In most articles, harmonization of data formats, data models, terminologies, ontologies, and analytical tools, as well as working practices were proposed as possible solutions to standardization issues [3–5,9,11,33,34,39]. A consensus of standards across the entire data flow [3–5,9,33,36], the effective use of datasets [5], data optimization [34], data consistency [38,49] and replicability of the studies [4,11] were also suggested as a means to foster data sharing. The adoption of unified data standards was considered to be important in both academic and industry settings [9,36].

To improve standardization efforts and data sharing, the systematic adoption of FAIR (Findable, Accessible, Interoperable, Reusable) Guiding Principles for scientific data management [51] was proposed [9]. Other authors mentioned the need for specialized organizations to promote harmonization of terminologies in health research [4,9]. An example is the consortium behind the Fast Health Interoperability Resources (FIHR) standard to enable “interoperable communication and information sharing between various healthcare systems” [9].

*3*. *Data quality and biases in data*

CHALLENGES: Data quality of unstructured data was frequently cited as an important challenge for evidence creation [4,5,8,11,18,34,37,38,40,50]. Unstructured data are often collected for purposes other than research and may lack systematic collection methods and scientific rigor, thus often leading to missing data [4,5,8,11,18,34,38,47]. In medical records, for example, missing data can occur because health care professionals may omit some information or because of patients’ refusal to share data [8]. The challenge of data quality is reinforced by data inconsistencies and inaccuracies [4,8,11,34,38,40]. Other recurrent challenges stem from biases in data collection—mainly in the form of selection and information bias [5,8,18,34,37–39]—and confounding [4,39]. Selection bias was mentioned, for example, in the context of studies where the sample comprised only of individuals who have the digital literacy skills or interest to share unstructured data from social media or wearable sensors [5,12]. Information bias, such as observer bias, was often mentioned in the context of making errors with data in EHRs use and big data analytics [8,37–39]. Further biases may establish themselves in analyses if processing algorithms were trained on biased data [18,38,39]. Finally, the quality and continuity of data might be negatively impacted by technical issues that can arise, for example, by software updates of wearable sensors [5].

POSSIBLE SOLUTIONS: Several strategies were proposed for assessing and ensuring data quality [3–5,8,18,38]. For studies that use digital health technologies, one study cited a recommendation from the European Medicines Agency (EMA) urging researchers to define “small, well-defined, meaningful measures followed by a data-driven development path” [5,52]. Furthermore, possible data quality issues should be considered for all study phases, including preprocessing, feature extraction or analysis [18]. First, preprocessing should yield only verified and valid dataset that properly combines the unstructured data with other data sources, for example by ensuring that study samples are representative of the populations that are being studied. Second, following feature extraction, data should be critically assessed for their validity and meaning. Finally, analytical methods should be aligned with the research goals of description, prediction, or prescription of the study in such a way that bias is reduced.

Other studies highlighted the need for a data quality standard checklist such as Data Access Quality and Curation for Observational Research Design (DAQCORD) [18,53]. This checklist should provide a priori guidance for planning of large-scale study data collection and pre-processing [54], thereby countering the pervasive practice of post-hoc methods for data cleaning [18]. Other proposed possible solutions included the use of meta-information to increase data quality, to detect potential biases in the data [3], to enable cross-referencing of multiple data sources involving the same individuals, as well as to encourage the comparison of results [4]. Imputation procedures for addressing missing data, as well as algorithms for checking data quality were also recommended [18]. Furthermore, the inclusion of study participant feedback can inform data collection and processing and improve the relevance of study findings for the intended target population [5].

*4*. *Infrastructure and Human Resources*

CHALLENGES: Several studies pointed out challenges related to infrastructure availability, including databases, or open-source platforms [3–5,8–10,18,33]. Infrastructure challenges can be particularly problematic when healthcare data are spread across multiple medical systems that lack connection or interoperability, thus creating isolated data clusters [9,10,33,36]. Difficulties in data linkage can also emerge when information system architectures cannot accommodate data standardization and other linkage processing tools [18]. Furthermore, the lack of skills and formal training opportunities for infrastructure utilization or inadequate knowledge of novel statistical tools and methods for combining unstructured data with other data sources can inhibit their use in health research [4,5,8].

POSSIBLE SOLUTIONS: Improvements such as searchable catalogues, databases and the availability of open platforms can mitigate infrastructure-related challenges [4,5,9,11,16,18,32,39]. Similarly, the availability of infrastructure for the storage and combination of unstructured data with other data sources can enable collaborative efforts, facilitate standardization, and foster the alignment of unstructured data with good research question development and research design [4]. Furthermore, the availability of secure collaborative platforms and repositories for data sharing through open science can enable independent knowledge gain and foster new research studies [32,34]. Meta-databases or catalogues that facilitate the discovery of open data and linking data across public repositories can also facilitate digital unstructured data enrichment [4,9]. Several studies further suggested that platforms for combining different datasets from various sources should have a modular, flexible, and scalable structure [9,18,32] and recommended to define the purpose and goals of such platforms during their development [11,39]. Open data and open software repositories also provide more opportunities for external validation of novel algorithms or (electronic) clinical outcome measures [16]. Finally, awareness about novel digital unstructured data enrichment methods, their methodological requirements, and the need for specialized training opportunities should be increased [4,11].

*5*. *Finding suitable analysis tools*, *methods*, *and techniques*

CHALLENGES: The complexity of analyses and appropriate methodological choices associated with the unstructured data enrichment in health research are challenges that were addressed in multiple studies [4,8,9,18,32,38,39]. Typical features of unstructured data such as high volume or complexity may be overwhelming for researchers due to a lack of methodological knowledge and might discourage researchers to use them in combination with other data sources for enrichment purposes [8]. Furthermore, the validity of results may be decreased by algorithms that are either not trained properly or may need recurrent fine-tuning to ensure that they create a model representative of its intended purpose and without biases [8,16,18]. Working with unstructured data requires specific expertise, typically from data scientists. However, the lack of supply of data scientists or the failure to build effective collaborations with external experts were also cited as impediments to managing the complexity of unstructured data [4,9,39]. Furthermore, there is a lack of guidelines and standards to guide decisions on which tools, methods, and analytical approaches to use when using unstructured data in health research [4,8].

We further observed a discrepancy in approaches to reduce the complexity of unstructured data (e.g., using feature extraction) in our studies. While some authors argued that complexity reduction is a feasible and appropriate method to enhance the combination of unstructured data with other data sources, others voiced concerns that complexity reduction can also reduce richness of unstructured data—particularly in the context of EHRs [13,32].

POSSIBLE SOLUTIONS: The complexity of unstructured data calls for increased collaboration among different experts. The increasing need for interdisciplinary efforts among health researchers, data scientists, biostatisticians, and health-care professionals was highlighted by most sources [3–5,11,16,32,34,37]. Some authors emphasized the need for a novel profession that combines expertise in health research and informatics [4,34]. Many also called for greater attention to trainings of health researchers regarding novel methods for using and combining unstructured data with other data sources [4,11,16]. The need for specific sets of skills, resources, and guidelines for the successful implementation of big data tools into clinical workflows was further mentioned as a requirement to manage unstructured data complexity [38]. Furthermore, some authors called for more efforts to develop and establish validated algorithms to process and integrate data [8]. One suggestion was to “provide AI with more ‘functional’ information, such as domain-specific medical reasoning processes and policies based on heuristic-driven search methods derived from human diagnostician methods” [9]. It was also suggested that the use of multiple data sources can improve the performance of ML models, such as through combination of theory-driven and data-driven approaches [50]. In the field of mental health, it was advised to complement data-driven research with qualitative research to strengthen the relevance and meaning of results [39].

*6*. *Alignment with a research design and/or research question*

CHALLENGES: The difficulty of finding suitable datasets and their subsequent, critical evaluation for clinical relevance was discussed from several perspectives [4,8,16,34,37–39]. One study strongly warned against adjusting the research agenda to the data that are available [39]. Furthermore, the fact that unstructured data or technologies generating these data were not designed for scientific purposes [11,16,37,39] might lead to misinterpretation of the data [39]. The lack of contextual (meta) information, for example about the data generation process, and observational nature of many sources of unstructured data may limit the value of the data for their use in robust, replicable confirmatory analyses (e.g., regarding disease etiology or intervention) [37,39]. The need for further and robust validation of results or outcomes from unstructured data analyses was a further topic of concern [11,38,39,50]. For example, predictive models need further validation before being integrated into clinical settings [38] and informing clinical decision-making [8]. Similarly, while linked EHRs are suitable for generating research questions, unstructured data should not be used for influencing clinical practice without prior validation [4].

POSSIBLE SOLUTIONS: It should be ensured that unstructured data used in combination with other data sets are relevant for a research question and desired therapeutic effect [16]. When working with data from digital health technologies, the EMA recommendation framework—that was developed with the collaboration with industry representatives with the aim to provide insights and guidance on validation and qualification processes of digital technologies [54]—could be consulted for guidance with research question design [5].

Another recommendation was to align large-scale research projects using unstructured data with clinical priorities and outcome-focused research [11]. Similarly, the choice of analytical tools depends on the goals of health research: description, prediction, or prescription [18]. Thus, setting clear research goals might help with the choice of appropriate analytical tools and methods. Finally, unstructured data should be used rather with complementary and enrichment purposes than as a replacement of other traditional methods or datasets [12,16,38,39].

*7*. *Ethics & Legal Issues*

CHALLENGES: The most frequently mentioned ethical challenges concerned privacy protection, informed consent and preservation of individual agency over data use [4,5,8–11,18,38,40]. Further challenges connected with digital unstructured data enrichment include inappropriate patient profiling [38] and decreased participants diversity due to low digital literacy skills reducing some participants’ contributions to certain types of unstructured data (e.g., from social media use) [5]. Furthermore, current deidentification and anonymization practices may still allow patient-linkage when combining different data sets. This is, for example, enabled when a combination of data on unusual physical conditions of a patient from a local hospital or a combination of gender, age and admission date might be unique enough to identify a subject and connect it with consumer-level data [13,18].

POSSIBLE SOLUTIONS: Strategies for preserving data privacy and security were discussed in multiple studies [4,5,8,10,11,16,18,43]. Some authors proposed to develop a new social contract and a broad consent model to balance the benefits of data usage and privacy concerns [4,8,11]. Unified rules for data governance across fields and sectors might contribute to systematic privacy protection and confidentiality [18], such as through unified procedures for data anonymization. Additionally, the importance of engagement with regulatory agencies in early stages of research was emphasized to ensure alignment of unstructured data processing with best practices [5]. Finally, independent agencies or governing bodies were proposed to oversee and ensure safe data sharing, preservation of intellectual property and valid applications [16,18].

## Additional recommendations

During the literature review, we identified two additional, overarching recommendations for unstructured data use, which are described subsequently.

### Collaborations with all stakeholders

Several sources stressed the importance of stakeholder collaboration in health research when combining different data sources for knowledge enrichment [5,9,11]. The inclusion of with public and patient advocacy groups and other relevant stakeholders was highly recommended [11] to ensure wide public acceptance and patient trust [4,43]. Broad stakeholder involvement was also seen as crucial to increase data sharing and to minimize wasted efforts from research study duplication [5]. Collaborative efforts among academic and commercial organizations (e.g., digital device or sensor manufacturers) can facilitate large-scale data integration and create synergies [9,11]. Stakeholder and patient engagement during in the digital unstructured data enrichment, analysis, and interpretation provides relevant context and feedback on the meaningfulness of results [5,11].

### Documentation and transparency

Proper documentation and transparency during the entire data flow were repeatedly mentioned as essential steps to achieve reliability, replicability, reproducibility and validity of studies, as well as facilitating the standardization efforts to ultimately enable unstructured data enrichment [5,9,37,39]. The EMA framework emphasizes documentation as an important means to achieve reliability, repeatability, accuracy, clinical validity, generalizability, and clinical applicability of the novel methodologies [5]. In the context of digital health technologies, United States Food and Drug Administration (FDA) recommendations suggest documenting the device and algorithm input and output, and to provide plans for data loss minimization, missing data handling, or patient inclusion for results. Furthermore, the FDA recommendations call for transparency of all processing steps from raw data to algorithm and at all data workflow stages [5]. Transparency regarding the analysis process can also assist with the assessment of whether study findings were clinically significant [39]. Specifically, studies relying on large databases will produce many statistically significant, but clinically meaningless results. This “overpowering” of statistical tests by large sample sizes should be made transparent through reporting of effect size determinants and complementation by clinical interpretation [37].

### Proposal for a feasibility and planning checklist for unstructured data enrichment

Many studies highlighted the need for further research and guideline development on best practices to use and integrate unstructured data in health research [4,9,11,16,33,34,38]. In Table 1, we provide a set of guiding questions to inform early study planning and the assessment of the feasibility of studies. These questions are based on the described challenge areas, which have been expanded to align with the breadth of proposed solutions from our review.

**Table 1:** The checklist for early study planning and the assessment of the feasibility of studies using digital unstructured data.

Key Issues	Comments
**Sufficient Metadata & Documentation for Unstructured Data** [3–5,18,43]	Meta-information can describe primary data and provide contextual information about data collection, pre-processing, or interpretation. Meta-information is especially important for data that were collected for purposes other than research or data from wearables and other electronic devices.
1. Is meta-information for the unstructured database available and where?	Meta-information should be findable and well documented.
2. Can meta-information offer sufficient contextual information for data interpretation?	Meta-information should include [3]: Person: e.g., subject ID, medical history, or demographics Context of collection: environment, study ID, or procedure description Observations: e.g., technology-affiliated site location, technology type, or notes made by an observer (e.g., a clinician) Time of data collection: e.g., time source, time zone, or medication schedule
**Standardization Options for Unstructured Data** [3–5,8–10,13,16,18,33,34,37,43,45,48,50]	
3. Are data transformable into a standardized format?	A standardized format can be a tabular format. Many different standards for clinical data already exist. For example, for EHRs, the Fast Healthcare Interoperability Resources might be useful [55].
4. Do data already contain standardized syntax/terminology or can such standards be applied?	Clinical information such as terminology and coding for diseases (e.g., ICD-10) might differ across databases. For data linkage, sharing and reproducibility, standardized syntax and terminology are important.
5. Does the dataset contain standardized semantics/ontology, or can such standards be applied?	Standardized ontology describes logical relations between core concepts to structure the description of data and foster interchangeability and consistency of data [49]. For example, Systematized Nomenclature of Medicine—Clinical Terms (SNOMED CT) is a comprehensive medical terminology used for electronic health data [4,56].
**Data Quality** [18,38,49,50]	For observational studies, the checklist DAQCORD [53] might be a useful starting point.
6. Can the consistency of data be secured? Are strategies/methods/steps available and included in the data management to secure consistency of data?	Consistency of data refers to the concept that the same data stored in separate places or separate time points still match, meaning contradictory conclusions cannot be derived from the given data [57]. For example, can be ensured that archived/backed-up/repository-deposited information can be kept up to date?
7. Is the dataset without a significant amount of missing data?	Due to selective reporting, EHRs may lack important data because clinicians did not deem them relevant, or patients did not want to share them [8].
8. Are strategies/methods available and/or defined for dealing with missing data?	Data may be missing for different reasons. They may be ‘missing at random’, they may be missing because the information was deemed irrelevant (e. g., not collected or not relevant for research question), because of branching or procedural logics (e.g., data are only collected under certain conditions). This knowledge also informs the feasibility of multiple imputation techniques, which assume at least some randomness (either systematic or non-systematic) in missing data.
**Data Validity** [3,8,11,18,50]	
9. Is the population for which the data are available representative of the target population?	Data might be only available for a limited population, for example., only a particular population group used the device collecting the data. Also, physicians’ notes are only available for a select subgroup (e.g., persons with a more severe clinical presentation). A sound understanding of the data generation process (possibly informed by meta-information) is essential.
10. Are strategies implemented to prevent or minimize the risk that the data are affected by selection or information biases?	Biases can prevent that the measures or outcomes correspond to their true value. Epidemiological and medical research commonly distinguishes between three types of biases [58]: Selection bias occurs when the selection of study participants alters the exposure-outcome relationship (not to be confused with external validity/representativeness). Information bias occurs when the ways of how data is collected impair data accuracy. Confounding refers to an observed relationship between exposure and outcome, which is influenced by a third, unaccounted variable (e.g., lung cancer is more prevalent among persons who drink alcohol, but smoking is also associated with alcohol consumption) [59]. Note that terminologies regarding biases may differ across scientific disciplines.
**Alignment with Research Question and Design** [5,12,16,38,39]	
11. What is the purpose or motivation of enriching a dataset with unstructured data?	For example, unstructured data can provide additional insights into individuals’ lived experiences or provide information in higher temporal resolution than standard data collection approaches (e.g., surveys).
12. Can the purpose of unstructured data be linked with a well-defined research question?	It is advisable to specify clearly defined and operationalized hypotheses before conceptualizing and conducting the study Considerations are needed whether unstructured data enrichment increases the chances for successfully testing of these pre-specified hypotheses.
13. Can the use of unstructured data be aligned with the planned research task (description, prediction, exploration, explanation, application)?	The aim of digital unstructured data enrichment might differ depending on research tasks, e.g., description (such as describing a disease progression), prediction (predicting outcomes), exploration (to find new patterns or generate a new hypothesis), explanation (to establish causality) or application (such as the development of a practical tool for diagnosis).
14. Can the combined dataset lead to relevant, novel insights?	It should be considered what added value can be expected by the enrichment with unstructured data. Examples are deepened qualitative or quantitative insights, more real-time time data, or a stronger participant-centeredness.
**Infrastructure for Processing and Analysis** [3–5,9,11,16,32,34,37,38]	
15. Does the research team have relevant skills/ or access to experts to approach digital unstructured data enrichment?	Interdisciplinary teams should include persons with strong (quantitative and/or qualitative) research methods skills as well as subject domain knowledge (e.g., specialists in a particular clinical area).
16. Can the interdisciplinary work be well established?	Define strategies to include persons with the necessary skills in the project teams, e.g., through existing networks, through consulting services (e.g., statisticians), referral by colleagues.
17. Can any duplication of research be excluded?	It is advisable to search for and summarize existing literature. It might be useful to check open data sources, platforms, database aggregators or searchable catalogues
**Availability of suitable analysis tools, methods and techniques** [4,8,9,11,16,39]	
18. Are the appropriate methods, tools, and techniques available?	The combination of unstructured data with other data sources requires a set of different methods, tools and techniques from informatics, data science, software development and others. The complexity of data opens many possibilities for analysis and statistical methods and their choice should be well justified.
19. Were the analysis methods, tools, and techniques chosen in a way that does not increase the risk of biases?	Analytical methods should ideally match the pre-specified study questions and hypotheses—not the other way around.
20. Can unstructured data be structured without significant loss of richness or other limitations? If no, will such limitations be reported/documented?	For example, qualitative information about patient experience from EHRs might get lost. Consider the integration of qualitative information as part of the analysis. Consider spot-checks and validation of quantitative findings using unstructured data (e.g., through random chart reviews).
**Expected quality of evidence of combined database** [4,5,8,11,18,34,37,38,40,50]	
21. Can the methodology of hypothesis testing be well defined?	Given a set of pre-specified hypotheses: Are the data and planned methods suitable to detect the effect of interest (as indicated by, e.g., an a priori power analysis)?
22. Can the results be sufficiently validated to serve as research evidence?	Validation means the testing of (prediction) models and study findings in other, previously unused data. Validation pertains to testing whether the study findings can be applied to other similar individuals outside of the study and whether a statistical relationship, for example between cause and effect, can be generalized [60].
23. Can unstructured data be technically combined/merged with structured data?	What are the links between structured and unstructured data? Are there shared unique identifiers in both databases? Do the unstructured data need matching by specific time-points? Common challenges are that structured and unstructured data are not collected at synchronized time points and/or for all participants.
24. Can input from patient/population from whom the data was collected be meaningfully included in the study?	Including qualitative data of individuals’ input about their experience, for example, with wearable sensors can provide important contextual information for ensuring quality and relevance of data and the study.
**Ethical and Legal Aspects** [4,5,8–11,16,18,43]	
25. Have ethical and data security requirements been clarified and reviewed? If not, is it planned to contact relative authorities and regulators be contacted to clarify privacy and ethical requirements?	Many studies involving health data require approval by ethics committees. Moreover, it may be advisable to seek contact with data protection officers upfront to assess and identify potential data security and privacy risks.
26. Have strategies for securing data privacy and security been clarified and are ready for implementation?	Linkage, processing, and analysis of unstructured and structured data require planning and consideration of the complete data life cycle. A data management plan should be put in place to outline rules and principles for handling data.
27. Can the data be fully anonymized or pseudonymized?	Deidentification efforts might not lead to full anonymization because an individual might be uniquely identified due to a specific piece or aggregates of information.
**Transparency, Reporting** [5,9,37,39]	
28. Can all the steps of data collection and management be well documented?	With the steps of data management, we mean data collection, data source, data storage, data retrieval, data preprocessing, data analysis, data interpretation. Ideally, these considerations should be included in a data management plan and cover the full data life cycle.
29. Can be ensured that the documentation contain elements of established reliability, accuracy and validity of the studies?	Reliability refers to the stability of findings [61]Accuracy is the proximity of measurement results to the "true" value. Validity is the truthfulness of findings; the results of an experiment do measure the concept being tested [62,63].
30. Is the analysis process being documented in a detailed fashion (e.g., inclusion of the description of analysis on the level of coding or data) that allows sharing and replication?	This is a requirement for open science. A study protocol can be a good starting point for documentation. Moreover, all preprocessing and analysis steps should be programmed/coded and commented on.
31. Can the documentation support generalizability and replicability of studies?	Replicability is obtaining consistent results across studies aimed at answering the same scientific question [64]. Generalizability means that the study results or outcomes are applicable also in other study settings or samples [65].
32. Can limitations of data be reported?	For example, it should be reported whether unstructured data was only collected from a limited group of population or the collected textual data from social media was limited to long posts which might lead to a collection of data from a population group with specific characteristics.
33. Can the relevant technical steps of the data linkage be reported?	This includes strategies, definitions and techniques for combining structured with unstructured data. For example, were data linked by person and for specific time points? What were assumptions and definitions used in the linkage process (e.g., was there a pre-specified time window within which two data points/assessments were considered as simultaneous)?
**Reproducibility of pre-processing, feature extraction and analysis, Open Science** [4,5,8,11,16,18,32,39]	Reproducibility means obtaining consistent results using the same input data; computational steps, methods, and code; and conditions of analysis. Reproducibility is closely connected with transparency, sufficient reporting and availability of data and methods [65]. For reproducibility purposes and data sharing, FAIR principles might be particularly useful. FAIR [51] means “Findability, Accessibility, Interoperability, and Reuse of digital assets”.
34. Can it be ensured that the preprocessing steps reproducibly yield valid intermediary/analytical data?	See points above regarding documentation (questions 28–33).
35. Can it be ensured that the feature extraction algorithms reproducibly yield data for meaningful statistical processing? Are extracted features sensitive to changes/adaptations in algorithms?	See points above regarding documentation. Documenting algorithms may be challenging when relying on proprietary software.
36. Can raw data / intermediary data / analytical data be made openly available?	Whether and how data can be made available depends, for example, on data ownership, the availability of informed consents by participants, privacy risks, risks for re-identification.
37. Can raw data / intermediary data / analytical data be integrated into a well-designed open platform repository?	Many open data repositories with different requirements regarding data format or documentation exist. Many scientific journals demand a mandatory upload of certain data types into public repositories (e.g., for genetic sequence data).

## Discussion

### Summary of findings

Our systematic narrative review provides an overview of challenges and best practices associated with the combination of unstructured data with other data sources in the fields of cardiology, neurology and mental health, which we refer to as digital unstructured data enrichment. In our review, we identified seven prevalent challenge areas in enabling digital unstructured data enrichment: 1) the lack of meta-information for unstructured data, 2) standardization issues, 3) data quality and bias in unstructured data, 4) infrastructure and human resources, 5) finding suitable analysis tools, methods and techniques, 6) alignment of unstructured data with a research question and design, as well as 7) legal and ethical issues. For each challenge area, we summarized proposed possible solutions. Additionally, we derived two additional recommendations that span across all challenge areas. We also compiled literature and experience-based checklist questions to inform initial study planning about the feasibility of research studies aiming to complement existing health data with digital unstructured data.

### Description of main requirements and solutions to enable digital unstructured data enrichment

All our studies revealed challenges of unstructured data use in health research, many of which might endanger scientific rigor and quality of health studies that may inhibit digital unstructured data enrichment. For example, the frequently unclear suitability of digital unstructured data to address concrete research questions or allow for proper research study design [4,8,16] may lead to possible biases, threatening the external and internal validity of studies. The validity of studies might also be endangered by applying unsuitable analytical tools and methods. Furthermore, the findings of the study may lack generalizability limiting its use to specific research tasks and questions (e.g., hypothesis-generation) [4,8,16,37]. The lack of meta-information might hinder a proper interpretation of the data and consequently limit their use for enrichment purposes. Further problems are that the data can be placed so centrally that any bias will be strongly reflected in the results. The most discussed challenge of standardization issues might hinder replicability and generalizability of research studies. Finally, ethical and legal issues, such as the risk of patient re-identification when disparate data sources are combined, pose additional challenges to digital unstructured data enrichment.

While many of the challenges to enable digital unstructured data enrichment are not specific to the use of unstructured data and are well known (e.g., data quality or standardization issues), other challenges, such as difficulties to align data with research questions or challenges pertaining to special skills or infrastructure needs, may be aggravated with the use of unstructured data for enrichment purposes due to their complexity. One of the key challenges might be the lack of open and collaborative platforms that can foster not only joint standardization but also validation efforts [3,4,9]. Oftentimes, the attractive characteristics of unstructured data that might add value to research are the ones that pose the most challenges. The data granularity and large, often international, population-based samples can enhance disease understanding or monitoring but also lead to methodological challenges, for example, regarding validity and choice of tools for analyses [8,39].

The review revealed that the possible solutions to enable digital unstructured data enrichment are less frequently and systematically discussed than the challenges. In particular, several sources discussed challenges without referring to the existing solutions or offering proposals for possible solutions. The difficulties with enabling digital unstructured data enrichment are also reflected in the fact that possible solutions may need to cover multiple challenge areas. Interdisciplinary collaboration, open science and transparency were the most mentioned possible solutions. Overall, the reported possible solutions and additional recommendations are important to sustain interchangeability, validity, reliability, generalizability, and reproducibility of studies.

### Requirement for guidance on and reporting of digital unstructured data enrichment

Our review also revealed that, despite the widespread use of unstructured data in health research, there is a lack of a systematic approach and guidelines for researchers to address challenges specific to digital unstructured data enrichment. Several of the selected articles acknowledged the need for more guidance [4,9,11,16,33,34,39], oversight or monitoring from agencies [5,13,16] and interdisciplinary teamwork and exchange to establish methodological approaches in the context of utilizing unstructured data in combination with other data sources in health research [4,8]. Only a few studies directly mentioned existing frameworks and standards such as EMA recommendations [5], FAIR principles [9], openHR [4] or DAQORD framework [18] in the context of unstructured data use. Recent efforts to provide guidelines are mainly focused either on a specific type of unstructured data or on specific challenges, for example, guidelines and standards for the use of social media data [61,66], guidelines regarding the use of EHRs [67,68], checklists and frameworks for evaluating the measurements made by digital technologies [69] or algorithms used for data analysis [70,71]. However, it is up for discussion whether these specific frameworks and guidelines are suitable to provide general guidance on challenges connected with digital unstructured data enrichment.

Our findings also reveal an underreporting of information relevant to digital unstructured data enrichment in health research. Even though digital unstructured data are used in combination with other data sources, the specific challenges connected with the linkage of these data sources are only sparsely reported. This is reflected in this review, where the reported challenges could be applicable to other types of data, such as big data, and to challenges of unstructured data without data enrichment. This rather general presentation of results points to the insufficient methodological guidance as well as reporting of challenges specific to digital unstructured data enrichment in currently available research. The lack of reporting of specific challenges and barriers of digital unstructured data enrichment could be addressed by providing researchers with reporting guidelines that include unstructured data-enriched methods. For example, current reporting guidelines such as STROBE [72] do not cover unstructured data-enriched analyses. Furthermore, the assessed studies rarely reported challenges relevant to combining unstructured data with other health data sources. Rather, the studies usually provided a description of the data collection and preprocessing steps, such as issues of noisy and missing data. However, they often lacked a description of data limitations or strategies to ensure high data quality for the analyses. In light of the growing volume of unstructured data in health research, experience sharing should be increasingly encouraged—either in published literature (e.g., also in appendices) or in other outlets. The lack of reporting and unavailability of guidelines not only hampers study reproducibility but also presents missed opportunities for learning and capacity building. Similarly to the guidance and research on the linkage of structured datasets,^11^ there is a need to provide more guidance and research on specific challenges connected with the linkage of digital unstructured data with other data sources and to assess their quality.

All this points to a growing need to define systematic ways of how to approach digital unstructured data enrichment in health research. This need is enhanced considering the interdisciplinary nature of studies working with unstructured data enrichment. The numerous challenges linked with unstructured data use or digital unstructured data enrichment should be reflected in systemic guidance on how to properly combine digital unstructured data with other data sources in health research. This can also facilitate interdisciplinary collaborations that are essential for digital unstructured data enrichment. Our review identified a special need for guidance to establish common standards to enable digital unstructured data enrichment to help researchers in the first stages of study planning and to assess the feasibility of studies combining unstructured data with other data sources [5,16,32]. The checklist derived from our review provides a first, pragmatic step towards classifying challenges and developing methodologies in health research involving digital unstructured data enrichment. In next steps, we hope to encourage specific research fields to dive deeper into our proposed checklist and adapt it to terminologies and issues that might be of a greater relevance in their respective research fields.

### Limitations

First, our definition of “digital unstructured data” may not be universal and definite. Further, although based on a systematic search and extraction process, we restricted our search to a few specific research fields due to the prevalent and growing use of unstructured data in these fields. We did not include books and book chapters and included only articles that mentioned the digital unstructured data enrichment already in the abstract. This might have led to the exclusion of articles that discussed the limitations of digital unstructured data enrichment in the discussion part. Therefore, our overview is likely not comprehensive. Furthermore, we a priori excluded imaging data and bioinformatics data from our literature search, which are an important source of unstructured data, but are often analyzed with highly specialized tools. For example, only considering various image acquisition methods and different techniques to handle noise in the image data would already add complexity that cannot be generalized to other unstructured data types [28,29]. In the systematic narrative review, we did not specifically discuss challenges and obstacles that are linked with learning algorithms used for unstructured data linkage or data analysis or interpretation. However, there is also precaution and guidance needed for choices about learning algorithms. Machine learning and deep learning algorithms are not immune to errors, biases and other limitations that can negatively impact validity, objectivity, and reproducibility of studies. Finally, the included studies predominantly reported challenges of unstructured data use in health research in the enrichment context. This observed underreporting limits our review results from describing specific challenges and solutions directly associated with digital unstructured data enrichment. However, we find that this is an important finding and calls for more efforts to report challenges and methods of digital unstructured data enrichment in health research.

## Conclusion

The combination of unstructured data with other data sources structured databases opens new avenues for more person-centered, contextualized, or more real-time analyses. However, multiple methodological and conceptual challenges demand attention, ideally even before an analysis is undertaken. A clear definition and focus on suitable study questions, interdisciplinary team-work, or transparent documentation and open science are key ingredients towards a more robust unstructured data enrichment methodology. Overall, our review also points to a need of more guidance—and possibly also standards for reporting results of digital unstructured data studies. Awareness should be raised among researchers to openly document encountered challenges and possible solutions in unstructured data enrichment projects to enable experience exchanges and learning. Moreover, existing reporting guidelines such as STROBE should consider adding specific instructions on the documentation of unstructured data enrichment processes.

## Supporting information

S1 Text Search Syntax. (PDF)

S2 Text The members’ list of Health Community of Digital Society Initiative, University of Zurich, Zurich, Switzerland. (PDF)

S1 Table Table 1. Description of the included studies. (DOCX)

S2 Table Table 2. Description of Challenge Areas. (DOCX)

S1 Data Data Extraction Sheet. (XLSX)

S1 PRISMA Checklist Preferred Reporting Items for Systematic reviews and Meta-Analyses extension for Scoping Reviews (PRISMA-ScR) Checklist. (DOCX)

## References

[1] Kong H-J. Managing Unstructured Big Data in Healthcare System. Healthcare informatics research. 2019;25: 1–2. doi: 10.4258/hir.2019.25.1.1 30788175 PMC6372467

[2] Unstructured Data—an overview | ScienceDirect Topics. [cited 21 Aug 2023]. https://www.sciencedirect.com/topics/computer-science/unstructured-data

[3] Badawy R, Hameed F, Bataille L, Little MA, Claes K, Saria S, et al. Metadata Concepts for Advancing the Use of Digital Health Technologies in Clinical Research. Digital biomarkers. 2019;3: 116–132. doi: 10.1159/000502951 32175520 PMC7046173

[4] Hemingway H, Asselbergs FW, Danesh J, Dobson R, Maniadakis N, Maggioni A, et al. Big data from electronic health records for early and late translational cardiovascular research: challenges and potential. European Heart Journal. 2018;39: 1481–1495. doi: 10.1093/eurheartj/ehx487 29370377 PMC6019015

[5] Stephenson D, Alexander R, Aggarwal V, Badawy R, Bain L, Bhatnagar R, et al. Precompetitive Consensus Building to Facilitate the Use of Digital Health Technologies to Support Parkinson Disease Drug Development through Regulatory Science. Digital biomarkers. 2020;4: 28–49. doi: 10.1159/000512500 33442579 PMC7768153

[6] Adnan K, Akbar R, Khor SW, Ali ABA. Role and Challenges of Unstructured Big Data in Healthcare. In: Sharma N, Chakrabarti A, Balas VE, editors. Data Management, Analytics and Innovation. Singapore: Springer; 2020. pp. 301–323. doi: 10.1007/978-981-32-9949-8_22

[7] Tayefi M, Ngo P, Chomutare T, Dalianis H, Salvi E, Budrionis A, et al. Challenges and opportunities beyond structured data in analysis of electronic health records. WIREs Computational Statistics. 2021;13. doi: 10.1002/wics.1549

[8] Silverio A, Cavallo P, de Rosa R, Galasso G. Big Health Data and Cardiovascular Diseases: A Challenge for Research, an Opportunity for Clinical Care. Frontiers in medicine. 2019;6: 36. doi: 10.3389/fmed.2019.00036 30873409 PMC6401640

[9] Termine A, Fabrizio C, Strafella C, Caputo V, Petrosini L, Caltagirone C, et al. Multi-Layer Picture of Neurodegenerative Diseases: Lessons from the Use of Big Data through Artificial Intelligence. Journal of personalized medicine. 2021;11. doi: 10.3390/jpm11040280 33917161 PMC8067806

[10] Shen B, Lin Y, Bi C, Zhou S, Bai Z, Zheng G, et al. Translational Informatics for Parkinson’s Disease: from Big Biomedical Data to Small Actionable Alterations. Genomics, proteomics & bioinformatics. 2019;17: 415–429. doi: 10.1016/j.gpb.2018.10.007 31786313 PMC6943761

[11] Hafferty JD, Smith DJ, McIntosh AM. Invited Commentary on Stewart and Davis \textquotedbl “Big data” in mental health research-current status and emerging possibilities\textquotedbl. Social psychiatry and psychiatric epidemiology. 2017;52: 127–129. doi: 10.1007/s00127-016-1294-4 27783131

[12] Andy AU, Guntuku SC, Adusumalli S, Asch DA, Groeneveld PW, Ungar LH, et al. Predicting Cardiovascular Risk Using Social Media Data: Performance Evaluation of Machine-Learning Models. JMIR cardio. 2021;5: e24473. doi: 10.2196/24473 33605888 PMC8411430

[13] Perera G, Broadbent M, Callard F, Chang C-K, Downs J, Dutta R, et al. Cohort profile of the South London and Maudsley NHS Foundation Trust Biomedical Research Centre (SLaM BRC) Case Register: current status and recent enhancement of an Electronic Mental Health Record-derived data resource. BMJ open. 2016;6: e008721. doi: 10.1136/bmjopen-2015-008721 26932138 PMC4785292

[14] Huckvale K, Venkatesh S, Christensen H. Toward clinical digital phenotyping: a timely opportunity to consider purpose, quality, and safety. NPJ digital medicine. 2019;2: 88. doi: 10.1038/s41746-019-0166-1 31508498 PMC6731256

[15] Zhang X, Pérez-Stable EJ, Bourne PE, Peprah E, Duru OK, Breen N, et al. Big Data Science: Opportunities and Challenges to Address Minority Health and Health Disparities in the 21st Century. Ethnicity & disease. 2017;27: 95–106. doi: 10.18865/ed.27.2.95 28439179 PMC5398183

[16] Espay AJ. Technology in Parkinson’s disease: Challenges and opportunities. Movement disorders: official journal of the Movement Disorder Society. 2016. doi: 10.1002/mds.26642 27125836 PMC5014594

[17] Sheikhalishahi S, Miotto R, Dudley JT, Lavelli A, Rinaldi F, Osmani V. Natural Language Processing of Clinical Notes on Chronic Diseases: Systematic Review. JMIR Medical Informatics. 2019;7: e12239. doi: 10.2196/12239 31066697 PMC6528438

[18] Foreman B. Neurocritical Care: Bench to Bedside (Eds. Claude Hemphill, Michael James) Integrating and Using Big Data in Neurocritical Care. Neurotherapeutics. 2020;17: 593–605. doi: 10.1007/s13311-020-00846-1 32152955 PMC7283405

[19] Succi S, Coveney PV. Big data: the end of the scientific method? Philosophical transactions Series A, Mathematical, physical, and engineering sciences. 2019;377: 20180145. doi: 10.1098/rsta.2018.0145 30967041 PMC6388004

[20] Harron KL, Doidge JC, Knight HE, Gilbert RE, Goldstein H, Cromwell DA, et al. A guide to evaluating linkage quality for the analysis of linked data. International Journal of Epidemiology. 2017;46: 1699–1710. doi: 10.1093/ije/dyx177 29025131 PMC5837697

[21] Sim I. Mobile Devices and Health. N Engl J Med. 2019;381: 956–968. doi: 10.1056/NEJMra1806949 31483966

[22] Hulsen T. Challenges and solutions for big data in personalized healthcare. 2021. pp. 69–94. doi: 10.1016/B978-0-12-822884-5.00016-7

[23] Kandel S, Heer J, Plaisant C, Kennedy J, Ham F, Henry Riche N, et al. Research directions in data wrangling: Visualizations and transformations for usable and credible data. Information Visualization. 2011;10: 271–288. doi: 10.1177/1473871611415994

[24] Park S, Lee SW, Han S, Cha M. Clustering Insomnia Patterns by Data From Wearable Devices: Algorithm Development and Validation Study. JMIR mHealth and uHealth. 2019;7: e14473. doi: 10.2196/14473 31804187 PMC6923760

[25] Ali F, El-Sappagh S, Islam SRiazulM, Ali A, Attique M, Imran M, et al. An intelligent healthcare monitoring framework using wearable sensors and social networking data. Future Generation Computer Systems. 2021;114: 23–43. doi: 10.1016/j.future.2020.07.047

[26] Dash S, Shakyawar SK, Sharma M, Kaushik S. Big data in healthcare: management, analysis and future prospects. Journal of Big Data. 2019;6. doi: 10.1186/s40537-019-0217-0

[27] Caliebe A, Leverkus F, Antes G, Krawczak M. Does big data require a methodological change in medical research? BMC Medical Research Methodology. 2019;19. doi: 10.1186/s12874-019-0774-0 31208367 PMC6580448

[28] Wang Y, Kung L, Gupta S, Ozdemir S. Leveraging Big Data Analytics to Improve Quality of Care in Healthcare Organizations: A Configurational Perspective. British Journal of Management. 2019;30: 362–388. doi: 10.1111/1467-8551.12332

[29] Kaur C, Garg U. Artificial intelligence techniques for cancer detection in medical image processing: A review. Materials Today: Proceedings. 2023;81: 806–809. doi: 10.1016/j.matpr.2021.04.241

[30] Page MJ, McKenzie JE, Bossuyt PM, Boutron I, Hoffmann TC, Mulrow CD, et al. The PRISMA 2020 statement: an updated guideline for reporting systematic reviews. BMJ (Clinical research ed). 2021;372: n71. doi: 10.1136/bmj.n71 33782057 PMC8005924

[31] Wissik T, &#270, ur&#269, O M. Research Data Workflows: From Research Data Lifecycle Models to Institutional Solutions. 2016 [cited 21 Aug 2023]. https://ep.liu.se/en/conference-article.aspx?series=&issue=123&Article_No=8

[32] Baldassano SN, Hill CE, Shankar A, Bernabei J, Khankhanian P, Litt B. Big data in status epilepticus. Epilepsy & behavior: E&B. 2019;101: 106457. doi: 10.1016/j.yebeh.2019.106457 31444029 PMC6944751

[33] Rodriguez A, Smielewski P, Rosenthal E, Moberg D. Medical Device Connectivity Challenges Outline the Technical Requirements and Standards For Promoting Big Data Research and Personalized Medicine in Neurocritical Care. Military medicine. 2018;183: 99–104. doi: 10.1093/milmed/usx146 29635618

[34] van den Heuvel L, Dorsey RR, Prainsack B, Post B, Stiggelbout AM, Meinders MJ, et al. Quadruple Decision Making for Parkinson’s Disease Patients: Combining Expert Opinion, Patient Preferences, Scientific Evidence, and Big Data Approaches to Reach Precision Medicine. J Parkinsons Dis. 2020;10: 223–231. doi: 10.3233/JPD-191712 31561387 PMC7029360

[35] Clark RA, Foote J, Versace VL, Brown A, Daniel M, Coffee NT, et al. The Keeping on Track Study: Exploring the Activity Levels and Utilization of Healthcare Services of Acute Coronary Syndrome (ACS) Patients in the First 30-Days after Discharge from Hospital. Medical sciences (Basel, Switzerland). 2019;7. doi: 10.3390/medsci7040061 31010168 PMC6524056

[36] Deferio JJ, Breitinger S, Khullar D, Sheth A, Pathak J. Social determinants of health in mental health care and research: a case for greater inclusion. Journal of the American Medical Informatics Association. 2019;26: 895–899. doi: 10.1093/jamia/ocz049 31329877 PMC6696493

[37] Blair LM. Publicly Available Data and Pediatric Mental Health: Leveraging Big Data to Answer Big Questions for Children. J Pediatr Health Care. 2016;30: 84–87. doi: 10.1016/j.pedhc.2015.08.001 26330268 PMC4685457

[38] Rumsfeld JS, Joynt KE, Maddox TM. Big data analytics to improve cardiovascular care: promise and challenges. Nature reviews Cardiology. 2016;13: 350–359. doi: 10.1038/nrcardio.2016.42 27009423

[39] Schofield P. Big data in mental health research—do the ns justify the means? Using large data-sets of electronic health records for mental health research. BJPsych bulletin. 2017;41: 129–132. doi: 10.1192/pb.bp.116.055053 28584647 PMC5451644

[40] Haines-Delmont A, Chahal G, Bruen AJ, Wall A, Khan CT, Sadashiv R, et al. Testing Suicide Risk Prediction Algorithms Using Phone Measurements With Patients in Acute Mental Health Settings: Feasibility Study. JMIR mHealth and uHealth. 2020;8: e15901. doi: 10.2196/15901 32442152 PMC7380988

[41] Jacobson NC, Summers B, Wilhelm S. Digital Biomarkers of Social Anxiety Severity: Digital Phenotyping Using Passive Smartphone Sensors. Journal of medical Internet research. 2020;22: e16875. doi: 10.2196/16875 32348284 PMC7293055

[42] Li B, Ding S, Song G, Li J, Zhang Q. Computer-Aided Diagnosis and Clinical Trials of Cardiovascular Diseases Based on Artificial Intelligence Technologies for Risk-Early Warning Model. Journal of medical systems. 2019;43: 228. doi: 10.1007/s10916-019-1346-x 31197490

[43] Papadopoulos A, Iakovakis D, Klingelhoefer L, Bostantjopoulou S, Chaudhuri KR, Kyritsis K, et al. Unobtrusive detection of Parkinson’s disease from multi-modal and in-the-wild sensor data using deep learning techniques. Scientific reports. 2020;10: 21370. doi: 10.1038/s41598-020-78418-8 33288807 PMC7721908

[44] Payrovnaziri SN, Barrett LA, Bis D, Bian J, He Z. Enhancing Prediction Models for One-Year Mortality in Patients with Acute Myocardial Infarction and Post Myocardial Infarction Syndrome. Studies in health technology and informatics. 2019;264: 273–277. doi: 10.3233/SHTI190226 31437928 PMC6785831

[45] Ross EG, Jung K, Dudley JT, Li L, Leeper NJ, Shah NH. Predicting Future Cardiovascular Events in Patients With Peripheral Artery Disease Using Electronic Health Record Data. Circulation Cardiovascular quality and outcomes. 2019;12: e004741. doi: 10.1161/CIRCOUTCOMES.118.004741 30857412 PMC6415677

[46] Sajal MSR, Ehsan MT, Vaidyanathan R, Wang S, Aziz T, Mamun KAA. Telemonitoring Parkinson’s disease using machine learning by combining tremor and voice analysis. Brain Inform. 2020;7: 12. doi: 10.1186/s40708-020-00113-1 33090328 PMC7579898

[47] Sükei E, Norbury A, Perez-Rodriguez MM, Olmos PM, Artés A. Predicting Emotional States Using Behavioral Markers Derived From Passively Sensed Data: Data-Driven Machine Learning Approach. JMIR mHealth and uHealth. 2021;9: e24465. doi: 10.2196/24465 33749612 PMC8088855

[48] Ahn I, Na W, Kwon O, Yang DH, Park G-M, Gwon H, et al. CardioNet: a manually curated database for artificial intelligence-based research on cardiovascular diseases. BMC medical informatics and decision making. 2021;21: 29. doi: 10.1186/s12911-021-01392-2 33509180 PMC7842077

[49] Matoba T, Kohro T, Fujita H, Nakayama M, Kiyosue A, Miyamoto Y, et al. Architecture of the Japan Ischemic Heart Disease Multimodal Prospective Data Acquisition for Precision Treatment (J-IMPACT) System. International heart journal. 2019;60: 264–270. doi: 10.1536/ihj.18-113 30799376

[50] Gillan CM, Rutledge RB. Smartphones and the Neuroscience of Mental Health. Annual Review of Neuroscience. 2021;44: 129–151. doi: 10.1146/annurev-neuro-101220-014053 33556250 PMC9107341

[51] FAIR Principles. In: GO FAIR [Internet]. [cited 21 Aug 2023]. https://www.go-fair.org/fair-principles/

[52] EMA. European Medicines Agency. In: European Medicines Agency [Internet]. [cited 22 Aug 2023]. https://www.ema.europa.eu/en

[53] Ercole A, Brinck V, George P, Hicks R, Huijben J, Jarrett M, et al. Guidelines for Data Acquisition, Quality and Curation for Observational Research Designs (DAQCORD). J Clin Trans Sci. 2020;4: 354–359. doi: 10.1017/cts.2020.24 33244417 PMC7681114

[54] Cerreta F, Ritzhaupt A, Metcalfe T, Askin S, Duarte J, Berntgen M, et al. Digital technologies for medicines: shaping a framework for success. Nat Rev Drug Discov. 2020;19: 573–574. doi: 10.1038/d41573-020-00080-6 32398879

[55] Index—FHIR v5.0.0. [cited 22 Aug 2023]. https://www.hl7.org/fhir/

[56] Home. In: SNOMED International [Internet]. [cited 22 Aug 2023]. https://www.snomed.org

[57] Shi P, Cui Y, Xu K, Zhang M, Ding L. Data Consistency Theory and Case Study for Scientific Big Data. Information. 2019;10: 137. doi: 10.3390/info10040137

[58] Delgado-Rodríguez M, Llorca J. Bias. J Epidemiol Community Health. 2004;58: 635–641. doi: 10.1136/jech.2003.008466 15252064 PMC1732856

[59] Freudenheim JL, Ritz J, Smith-Warner SA, Albanes D, Bandera EV, van den Brandt PA, et al. Alcohol consumption and risk of lung cancer: a pooled analysis of cohort studies. Am J Clin Nutr. 2005;82: 657–667. doi: 10.1093/ajcn.82.3.657 16155281

[60] Altman DG, Vergouwe Y, Royston P, Moons KGM. Prognosis and prognostic research: validating a prognostic model. BMJ. 2009;338: b605. doi: 10.1136/bmj.b605 19477892

[61] D’Souza RS, Hooten WM, Murad MH. A Proposed Approach for Conducting Studies That Use Data From Social Media Platforms. Mayo Clinic proceedings. 2021;96: 2218–2229. doi: 10.1016/j.mayocp.2021.02.010 34353473

[62] Ranstam J. Methodological note: accuracy, precision, and validity. Acta radiologica (Stockholm, Sweden: 1987). 2008;49: 105–106. doi: 10.1080/02841850701772706 18210319

[63] Trajković G. Measurement: Accuracy and Precision, Reliability and ValidityMeasurement: accuracy and precision, reliability and validity. In: Kirch W, editor. Encyclopedia of Public Health. Dordrecht: Springer Netherlands; 2008. pp. 888–892. doi: 10.1007/978-1-4020-5614-7_2081

[64] Reproducibility and Replicability in Science. Washington (DC): National Academies Press (US); 2019. 31596559

[65] Kukull WA, Ganguli M. Generalizability: the trees, the forest, and the low-hanging fruit. Neurology. 2012;78: 1886–1891. doi: 10.1212/WNL.0b013e318258f812 22665145 PMC3369519

[66] Kim Y, Huang J, Emery S. Garbage in, Garbage Out: Data Collection, Quality Assessment and Reporting Standards for Social Media Data Use in Health Research, Infodemiology and Digital Disease Detection. Journal of medical Internet research. 2016;18: e41. doi: 10.2196/jmir.4738 26920122 PMC4788740

[67] Beale SH Thomas. openEHR-Home. [cited 22 Aug 2023]. https://openehr.org/

[68] Jones KH, Ford EM, Lea N, Griffiths LJ, Hassan L, Heys S, et al. Toward the Development of Data Governance Standards for Using Clinical Free-Text Data in Health Research: Position Paper. Journal of medical Internet research. 2020;22: e16760. doi: 10.2196/16760 32597785 PMC7367542

[69] Digital Medicine Society (DiMe)—Advancing digital medicine to optimize human health. [cited 22 Aug 2023]. https://dimesociety.org/

[70] Bradway M, Gabarron E, Johansen M, Zanaboni P, Jardim P, Joakimsen R, et al. Methods and Measures Used to Evaluate Patient-Operated Mobile Health Interventions: Scoping Literature Review. JMIR mHealth and uHealth. 2020;8: e16814. doi: 10.2196/16814 32352394 PMC7226051

[71] van de Leur RR, Boonstra MJ, Bagheri A, Roudijk RW, Sammani A, Taha K, et al. Big Data and Artificial Intelligence: Opportunities and Threats in Electrophysiology. Arrhythmia & electrophysiology review. 2020;9: 146–154. doi: 10.15420/aer.2020.26 33240510 PMC7675143

[72] STROBE. In: STROBE [Internet]. [cited 22 Aug 2023]. https://www.strobe-statement.org/

