# Supplementary material for: Challenges and best practices for digital unstructured data enrichment in health research: A systematic narrative review
Source: PLOS Digit Health. 2023 Oct 11;2(10):e0000347. doi: 10.1371/journal.pdig.0000347 (PMC10566734; doi:10.1371/journal.pdig.0000347)
Supplement: S1 Text — (PDF) [file pdig.0000347.s001.pdf]

Supplementary information 1. Search Syntax

(("unstructured data"[Title/Abstract] OR "big data"[Title/Abstract] OR "multimodal data"[Title/Abstract] OR "text data"[Title/Abstract] OR "textual data"[Title/Abstract] OR "voice data"[Title/Abstract] OR "sensor data"[Title/Abstract] OR "video data"[Title/Abstract] OR "social media data"[Title/Abstract] OR "speech data"[Title/Abstract] OR "wearable data"[Title/Abstract] OR "sensing data"[Title/Abstract] OR "free text"[Title/Abstract] OR "patient narrative"[Title/Abstract] OR "natural language processing"[Title/Abstract]) AND ("health*"[Title/Abstract] OR "medical"[Title/Abstract] OR "clinical"[Title/Abstract] OR "patient"[Title/Abstract] OR "mHealth"[Title/Abstract] OR "digital health"[Title/Abstract] OR "patient-generated"[Title/Abstract] OR "PGHD"[Title/Abstract] OR "patient-reported"[Title/Abstract])) AND (("cardiovascular" OR "atrial fibr*" OR "myocardial infarction" OR "cardiolog*" OR "depression" OR "anxiety" OR "mental health" OR "mental disorder*" OR "neurolog*" OR "multiple sclerosis" OR "MS") AND ("integrat*" OR "appl*" OR "enrich*" OR "combin*" OR "harmoniz*" OR "synth*" OR "adopt*" OR "link*" OR "fusion") AND ("problem" OR "challeng*" OR "difficult*" OR "obstacle" OR "gap" OR "need" OR "issue*" OR "barrier*" OR "requirement*" OR "limitation*" OR "incompatib*"))
