# Supplementary material for: Challenges and best practices for digital unstructured data enrichment in health research: A systematic narrative review
Source: PLOS Digit Health. 2023 Oct 11;2(10):e0000347. doi: 10.1371/journal.pdig.0000347 (PMC10566734; doi:10.1371/journal.pdig.0000347)
Supplement: S1 Table — (DOCX) [file pdig.0000347.s003.docx]

S1 Table. Table 1. Description of the included studies

| **First author, year of**  **publication** | **Type of paper** | **Field (subfield)** | **Motivation for using unstructured data** | **Type of unstructured and structured data being integrated** |
| --- | --- | --- | --- | --- |
| Badawy R., 2019 | General (Review) | Neurology (Parkinson’s disease) | - Information enrichment: insights into patients’ symptoms, disease progression, and treatment efficacy - Cost effective and efficient - Remote, long-term monitoring in naturalistic settings - Increased statistical power of clinical trials - Reduction random sampling issues | Data from digital health technologies; focus on metadata |
| Baldassano S.N.,  2019 | General (Review) | Neurology (Status Epilepticus) | - Personalized medicine - Patients’ stratification for prognostication - Patients’ identification for interventions | Big data, more specifically: EHRs, administrative databases (e.g., data collected for billing purposes), iEEG, multimodal ICU data, physiological data (e.g., heart rate, respiratory rate) |
| Blair L. M., 2016 | General (Overview) | Mental Health (Pediatrics) | - Ease of access - Nationally representative sampling - Efficiency and lower cost | Publicly available big data |
| Deferio J.J. 2019 | General (Perspective) | Mental Health | - Richer contextual information - More robust set of patients social variables - Identification of social correlates of health outcomes | Data related to social determinants of health  EHR |
| Espay A. J., 2016 | General (Review) | Neurology (Parkinson’s Disease) | - Information enrichment - Treatment optimization - Maximization of “ecological” validity - Monitoring - High resolution descriptions - Quality of care - Personalized medicine | Data from health technology (e.g., wearable devices) |

| Foreman B., 2020 | General (Review) | Neurology (Neurocriticial Care) | - Patient cohorts’ enrichment - Population-level insights - Precision medicine - Evaluation of interventions - Cost efficient - Precision medicine | ICU data, EEG, unstructured free-text clinician notes, Digital Imaging and Communications in Medicine standard imaging, Logical Observation Identifiers Names and Codes (LOINC), standard laboratory values |
| --- | --- | --- | --- | --- |
| Gillan C. M. 2021 | General (overview) | Mental Health | - Higher-order level of analysis - Dense sampling - Repeated-within subject measurements - Data collection in naturalistic setting - Longitudinal data - Prediction - Better understanding of mental illnesses | Smartphone data Social media data  Structured data from neuroscientific research/neural measurements(e.g., fMRI, EEG) |
| Hafferty J. D., 2017 | General (Commentary) | Mental Health | - Naturalistic clinical settings - Analysis of rarer clinical conditions or subject areas - Prediction - Personalised medicine research - Better modelling of the ‘bio-psycho- social’ outcomes of psychiatric illness | Big Data in general |
| Hemingway H.,  2017 | General (Overview) | Cardiology | - High resolution results and large-scale studies - Real time analytics - Treatment improvement - More efficient and cost-effective methods | EHRs (structured and unstructured data); biobanks, genomic consortia; any researcher-generated data (e.g., omics data) |
| Rodriguez A., 2018 | General (Overview) | Neurology (Neurocritical care) | - Better patients’ stratification and management - High resolution descriptions | Genomic, imaging, physiological, and phenotypic  information (e.g., EEG), pre- and post- psychometric testing, EHR |

| Rumsfeld J. S., 2016 | General (Review) | Cardiology | - Prediction - Disease detection - Monitoring - Providing high resolution descriptions and large-scale studies - Prescriptive analytics - Quality of care and performance measures - Public health improvement | Big Data in general |
| --- | --- | --- | --- | --- |
| Schofield P., 2017 | General (Editoria l) | Mental Health | - Information enrichment: data about hard- to-reach groups, new relations between clinical outcomes and other factors | EHRs, data from other databases (e.g., hospital episode statistics, educational data) |
| Shen B., 2018 | General (Review) | Neurology (Parkinson’s disease) | - Screening - Holistic descriptions - Information enrichment | EHR, physiological data (EEG, electrocardiogram), omics data, neuroimaging data, epidemiologicaldata |
| Silverio A. 2019 | General (Overvie w) | Cardiology | - Insights into patients’ life, behavior, and symptoms - High resolution descriptions and large- scale studies - Monitoring - Public health improvement - Prediction - Personalized medicine - Quality of care and performance measures - Genetic insights | ‘Big Data’ in general |
| Stephenson D., 2020 | General (Review) | Neurology (Parkinson disease) | - Evaluation and subsequent approval of novel treatments - Assessment of aspects of the disease - Improvement of outcome measures - Better patient enrolment and stratification - Monitoring - Real-world data - Implementation of remote trials | Data from broad range of digital health technologies (e.g., smartphone applications, wearable sensors, GPS, EEG, digital diaries) |

| Termine A., 2021 | General (Review) | Neurology (neurodegenerati ve disorders such as Alzheimer’s  and Parkinson’s diseases) | - High resolution descriptions - Accuracy of analytical results - Quality of care - Prediction - Personalized and precision medicine - More efficient and cost-effective | EHRs, multi-omics, neuroimaging and wearable sensors data |
| --- | --- | --- | --- | --- |
| van den Heuvel L., 2020 | General | Neurology (Parkinson’s disease) | - Information enrichment - Real-time data - Personalized precision medicine and decision-making process | Big data in general |
| Andy A. U., 2021 | Research article | Cardiology (atherosclerotic cardiovascular disease) | - Prediction of individual risks - Better understanding of individual risks - Precision medicine | Facebook status updates, medicalrecord data |
| Clark R. A., 2019 | Research article | Cardiology (Acute coronary syndrome) | - Real world assessment | Quantitative and digital data  collection (GPS and GIS), qualitative patient-reported experiences (satisfaction survey), a  discharge education recallquestionnaire |
| Haines- Delmont A., 2020 | Research article | Mental Health (Suicide Prevention) | - Exploration - Prediction - Real-time, context-related monitoring - Ecologically momentary assessment in naturalistic settings - Real-time classification - Quantification of human behavior | Facebook, user data, clinician ratings, passive sensor data |

| Jacobson N.C., 2020 | Research article | Mental Health (Social Anxiety) | - Prediction - Monitoring | Social Interaction Anxiety  Scale; Depression, Anxiety, and Stress Scale; Positive Affect Negative Affect Schedule; passive sensor data (accelerometer data), incoming and outgoing calls and text timestamps |
| --- | --- | --- | --- | --- |
| Li B., 2019 | Research article | Cardiology (cardiovascular disease) | - Disease diagnostic - Early-risk prediction | Medical big data, medical diagnostic records |
| Papadopoulos A.,  2020 | Research article | Neurology (Parkinson’s disease) | - Unobtrusive, remote detection of early symptoms - Real-time data | Passively captured data from smartphones: the tri-axial acceleration values obtained from the  Inertial Measurement Unit sensor of the  smartphone and the keystroke timing data (press and release timestamps of each keystroke capturedduring typing with the smartphone’s virtual keyboard) |
| Payrovnaziri S. N.,  2019 | Research article | Cardiology (Acute myocardial infarction, post myocardial infarction syndrome) | - Prediction - Effective and efficient - Precision medicine - Quality of care - High resolution descriptions | Demographic and admission data (from EHR), free text from dischargesummary |
| Ross E. G., 2019 | Research article | Cardiology (periphery artery disease) | - Risk prediction of local populations or more specific disease states - Automatization of risk stratification | International Classification  of Diseases, Ninth Revision (ICD-9) codes, Current Procedural  Terminology codes, lab test values, prescription medications,  vital signs, unstructured  clinical notes |
| Sajal S. R., 2020 | Research article | Neurology (Parkinson’s diseases) | - Increased accuracy - Understanding of disease history, progression, and risk factors | Rest tremor and vowel phonation data acquired by smartphones |

| Sükei E., 2021 | Research article | Mental health | - Prediction of mood states - Assessment of behavioral features | Passively sensed behavioral data from6 sources, users’ record information |
| --- | --- | --- | --- | --- |
| Ahn I., 2021 | Paper on Database s | Cardiology | - Early-risk prediction - Discovery of risk factors and detection of their interactions - Prevention - Improvement of treatment planning | Structured data from electronic health records: demographics, vital signs, medication, laboratory test results, patient history questionnaire.  Unstructured data from text readings of electrocardiogram, coronary artery computed tomography, single-photon emission computed tomography |
| Matoba T., 2018 | Paper on Database s | Cardiology | - Real-time data - Precision medicine - Complementation to randomized clinical trials | Medical records, electronic  data regarding medical activity and procedures; key data regarding coronary angiography and percutaneous coronary intervention |
| Perera G., 2016 | Paper on Database s | Mental health | - Information enrichment - Novel investigations - Detection of patterns in patient care and treatment habits - Enhancement of research question | EHRs, diverse databases |
