# Supplementary material for: Challenges and best practices for digital unstructured data enrichment in health research: A systematic narrative review
Source: PLOS Digit Health. 2023 Oct 11;2(10):e0000347. doi: 10.1371/journal.pdig.0000347 (PMC10566734; doi:10.1371/journal.pdig.0000347)
Supplement: S2 Table — (DOCX) [file pdig.0000347.s004.docx]

S2 Table. Table 2. Description of Challenge Areas.

| **Challenge area** | **Definition/Description** | **Relevance** |
| --- | --- | --- |
| **1. Lack of meta- information for unstructured data** | All topics related to documentation of size, content, context, and format of unstructured data are included in this challenge area. | - To *scrutinize* and possibly *avoid biased* assumptions about the unstructured data (Silverio et al., 2019). - To provide *contextual information* for data interpretation (e.g., when and where data from wearable sensors were generated). The contextual information can enable a more *robust analysis* and *meaningful data interpretation* which is important to *evaluate* the *accuracy* of the unstructured data *and* its *interpretability* (Silverio et al., 2019; Badawy et al., 2019). - To facilitate the evaluation of the *quality and reliability* of the unstructured data (Badawy et al., 2019). - To facilitate replicability of studies. - To facilitate the *interchangeability and reuse* of unstructured data (Stephenson et al., 2020; Blair et al., 2016). - To facilitate the process of *validation for regulatory acceptance* (e.g., in the context of digital health technologies) (Stephenson et al., 2020). |
| 2. **Standardization Issues** | All challenges related to the conversion of unstructured data into a common standardized format that enables it to get shared, linked and used across different settings are included in this challenge area. | - To facilitate/foster effective data access, reuse of data for research projects and ultimately interoperability, interchangeability, and linkage of data (Termine et al., 2021; Stephenson et al., 2020; Ahn et al., 2020; Matoba et al., 2018). - To avoid duplication of research (Stephenson et al., 2020). - To facilitate data interpretation and extraction of correct information (Hemingway et al., 2017; Rumsfeld et al., 2016; Rodriguez et al., 2018; Schofield et al., 2017; Perera et al., 2016). - To enable data consistency (Rumsfeld et al., 2016). |

| **3. Data Quality and Bias in Data** | All topics concerning data accuracy, reliability, validity, and consistency are included in this challenge area. | - To increase/secure data accuracy, reliability, validity, reproducibility, replicability and consistency. - To avoid a loss of efficiency in achieving study goals, errors in analysis. - To facilitate interpretation of findings. |
| --- | --- | --- |
| **4. Infrastructure** | All topics related to IT infrastructure that enable or facilitate data management, access, sharing, and processing are included in this challenge area. | - To facilitate access to data that researchers need for their research (Hemingway et al., 2017; Stephenson et al., 2020). - To avoid missed opportunities by lack of accessibility to relevant data sources. - To reduce research costs (Termine et al., 2021; Rodriguez et al., 2018; Clark et al., 2019). |
| **5. Finding suitable analysis tools, methods, and techniques** | All topics related to the methodological choices of how unstructured data are processed and analyzed are included in this challenge area. | - To facilitate the complex process of cleaning and analyses of large and complex datasets (Silverio et al., 2019, Hemingway et al., 2017; Rumsfeld et al., 2016; Termine et al., 2021; Baldassano et al., 2019; Foreman et al. 2020; Schofield et al., 2017). - To decrease risk for bias in research (Foreman et al., 2020; Schofield et al., 2017). - To increase/secure data accuracy, reliability, validity, reproducibility, replicability and consistency. |
| **6. Alignment with a research design and/or research question** | All topics related to the broader theoretical issues of how the use and/or integration of unstructured data is linked with an appropriate research design and question is included in this challenge area. | - To ensure scientific rigor and validity (Stephenson et al., 2020). - To determine the most suitable data analysis approach (Badawy et al., 2019). |
| **7. Ethics & Legal Issues** | All topics emerging from ethical and legal concerns or risks either on the societal or individual level – such as privacy, confidentiality, safety, and discrimination – are included in this challenge area. | - Adherence to ethical and legal frameworks is a condition sine que non for research and requires no further justification. - To facilitate a successful integration of unstructured data in health research as it can increase public trust and acceptance which might lead to increased availability of data sources. |
